# Improving the Transparency of Meta Analyses with Interactive Web Applications

**DOI:** 10.1136/bmjebm-2019-111308

**Published:** 2020-03-27

**Authors:** Thomas P Ahern, Richard L. MacLehose, Laura L. Haines, Deirdre P. Cronin-Fenton, Per Damkier, Lindsay J. Collin, Timothy L. Lash

**Affiliations:** (1) Departments of Surgery and Biochemistry, Larner College of Medicine, University of Vermont, Burlington, Vermont; (2) Division of Epidemiology and Community Health, University of Minnesota School of Public Health, Minneapolis, Minnesota; (3) Dana Medical Library, Larner College of Medicine, University of Vermont, Burlington, Vermont; (4) Department of Clinical Epidemiology, Aarhus University Hospital, Aarhus, Denmark; (5) Department of Clinical Chemistry and Pharmacology, Odense University Hospital, Odense, Denmark; (6) Department of Clinical Research, University of Southern Denmark, Odense, Denmark; (7) Department of Epidemiology, Rollins School of Public Health, Emory University, Atlanta, Georgia

**Keywords:** Meta-Analysis as Topic, Reproducibility of Results, Consensus Development, Bayes Theorem, Breast Neoplasms, Tamoxifen

## Abstract

Increased transparency in study design and analysis is one proposed solution to the perceived reproducibility crisis facing science. Systematic review and meta-analysis—through which individual studies on a specific association are ascertained, assessed for quality, and quantitatively combined—is a critical process for building consensus in medical research. However, the conventional publication model creates static evidence summaries that force the quality assessment criteria and analytic choices of a small number of authors onto all stakeholders, some of whom will have different views on the quality assessment and key features of the analysis. This leads to discordant inferences from meta-analysis results and delayed arrival at consensus. We propose a shift to interactive meta-analysis, through which stakeholders can take control of the evidence synthesis using their own quality criteria and preferred analytic approach—including the option to incorporate prior information on the association in question—to reveal how their summary estimate differs from that reported by the original analysts. We demonstrate this concept using a web-based meta-analysis of the association between genetic variation in a key tamoxifen-metabolizing enzyme and breast cancer recurrence in tamoxifen-treated women. We argue that interactive meta-analyses would speed consensus-building to the degree that they reveal invariance of inferences to different study selection and analysis criteria. On the other hand, when inferences are found to differ substantially as a function of these choices, the disparities highlight where future research resources should be invested to resolve lingering sources of disagreement.

## INTRODUCTION

Ninety percent of scientists surveyed by the journal *Nature* agreed that there is a reproducibility crisis facing science.^1^ Among the proposed solutions to the perceived crisis is increasing the transparency with which scientific research is conducted and reported.^2^ In clinical research, opportunities for increasing transparency include pre-registration of planned analyses and publicly posting the data sets and analytic code that produced published results. For example, when reporting an adjusted estimate of association between an exposure and outcome, authors present results from a modeling approach they deemed plausible for yielding an unconfounded estimate. Inevitably, some of the paper’s readers will disagree with their choice of statistical model, the set of variables for which the association was adjusted, and how specific variables were operationalized in models—any of which may challenge the validity of the reported association. In such scenarios, access to the original data and statistical code allow independent re-analysis to address potential limitations in published research. Transparency of this sort is expected to speed the process of consensus building in science and reduce the apparent disagreement in the conclusions of related studies.^2,3^ To this end, there have been prominent calls for data sharing,^3,4^ but the promise of this approach is hampered by realities imposed by data privacy concerns.

### Meta-analysis as a model scenario for poor transparency

Issues surrounding scientific reproducibility and transparency are showcased in the context of systematic review and meta-analysis—the process by which the set of studies of a specific exposure-outcome association are extracted from the literature, critically evaluated, and (if appropriate) quantitatively combined to yield succinct summaries of evidence.^5^ Meta-analyses are an essential conduit for dissemination of clinical research findings,^6^ especially now that the rate of publication on a given topic often outpaces the capacity of busy stakeholders to read, evaluate, and synthesize the literature on their own. Lately, the publication rate for meta-analyses has increased disproportionately to the publication rate for all medical literature.^7–9^ This rise may be attributable to a high frequency of redundant meta-analyses on a specific topic, to absence of administrative and financial barriers to conducting meta-analyses, and to the relative ease with which meta-analyses can be performed in an academic climate of publication-centric incentives for career advancement.^7,9^ Furthermore, rampant production of meta-analyses is associated with low-quality and biased reports, as illustrated by their apparent use as a promotional medium for special interests.^9–14^

Conventional (frequentist) meta-analyses assign a weight to each included study (*e.g.*, in proportion to each study’s precision), but the choice of which studies to include is left to the authors’ assessment of the strengths and weaknesses of candidate studies collected from the literature. Under this conventional approach, readers of meta-analyses are at the mercy of the authors’ discretion—though their own assessment of study quality may have differed substantially, had they been presented with the entirety of the evidence base. As in the regression example above, the meta-analytic result that a given reader would have arrived at, subject to their own training and judgment, may differ substantially from the published result. To improve the transparency and applicability of this process, we propose herein a novel, interactive approach to the conduct and dissemination of meta-analyses. Our approach promotes transparency in both conduct and reporting of meta-analyses, and we illustrate it using an ongoing, controversial topic from the breast oncology literature: the association between *CYP2D6* gene variants and breast cancer recurrence among women receiving tamoxifen therapy.^15^

### Example topic: tamoxifen pharmacogenetics

Tamoxifen, a selective estrogen receptor modulator (SERM), has been prescribed for decades to prevent recurrence of estrogen receptor (ER)-positive breast cancer.^16^ It accomplishes this by binding ER on breast tumour cells, blocking its activation by estrogens and subsequent growth signaling. While tamoxifen is capable of binding and blocking ER in its native chemical form, it is also oxidized in the liver into several metabolites with higher ER affinity. These metabolites are presumed to exert a substantial proportion of tamoxifen’s pharmacological effect. The cytochrome P450 (CYP) enzymes catalyzing the oxidation reactions are highly polymorphic, and some variant alleles yield less-active or completely inactive enzyme products compared with wild-type alleles. While five years of tamoxifen treatment cuts the rate of breast cancer recurrence approximately in half,^17^ treatment fails in a substantial number of patients. Women who carry reduced-activity CYP alleles may produce insufficient levels of the most-active tamoxifen metabolites, and may therefore have higher rates of breast cancer recurrence on therapy. Identification of host or tumour biomarkers to predict tamoxifen failure has been a research priority. Much of this research has focused on CYP2D6, a dominant CYP enzyme in the tamoxifen metabolic pathway. The *CYP2D6* gene is currently known to carry more than 100 well-characterized variant alleles.^18,19^ About forty of these alleles are associated with reduced activity or non-functioning enzymes, but most epidemiologic research has focused on the two most prevalent detrimental alleles, *4 and *10. The CYP2D6*4 allele (rs3892097) is observed in about one-fifth of people with European ancestry and encodes an inactive enzyme. The CYP2D6*10 allele (rs1065852) is observed in about one-third of people with Asian ancestry and encodes a reduced-activity enzyme.

### Epidemiologic evidence

Most tamoxifen pharmacoepidemiology studies have measured associations between *CYP2D6* variants and the rate of breast cancer recurrence or breast cancer-specific mortality in tamoxifen-treated women. There are important considerations to bear in mind when assessing the evidence contributed by these studies. The most important of these are variations in source population (primarily Asian versus Caucasian)^20^, sets of *CYP2D6* alleles assayed (*4, *10, and others)^21^, and source of DNA used for genotyping (tumour tissue *vs*. non-neoplastic tissue)^22^ –all of which could influence the magnitude and/or validity of study results. Furthermore, prior knowledge of tamoxifen pharmacology and efficacy places an upper limit on the expected relative risk associating variant alleles with recurrence.^23^ We explain each of these in turn.

#### Consideration #1: Heterogeneity according to study population and *CYP2D6* alleles

Effect estimates are expected to vary by CYP2D6 functional status, which is informed by the source population and the set of *CYP2D6* allele(s) used to define metabolizer status. From the underlying biological rationale, carriers of the *4 allele (most prevalent in Caucasians) are expected to have a higher rate of recurrence than carriers of the *10 allele (most prevalent in Asians) since the *4 variant completely eliminates enzyme function, while the *10 allele merely attenuates enzyme function. Relative risks associating *CYP2D6* variants with recurrence should therefore be higher magnitude in Caucasian/*4 studies than in Asian/*10 studies.

#### Consideration #2: Heterogeneity due to DNA source

DNA source may affect study estimates. Recall that it is hepatic CYP2D6 that catalyzes the oxidation of tamoxifen, and so germline genotype—not tumour genotype—is the etiologically important exposure to measure. Tumour-derived DNA may cause misclassification of germline genotype due to loss of heterozygosity (LOH), when one copy of the gene is deleted as a consequence of genetic changes in the induction phase of the tumour.^22^ Relative risks from studies that used tumour DNA may therefore have an expected bias toward the null, since heterozygotes would be more likely misclassified as homozygous for the higher-prevalence wild-type allele.

#### Consideration #3: A biological limit on the magnitude of association

Another important consideration is that the relative risk associating CYP2D6 function and breast cancer recurrence among those on tamoxifen has an upper limit of approximately 2.^22,23^ This follows from the well-established effect of tamoxifen treatment, which is to reduce the recurrence rate by about half,^17,23^ and the supposition that tamoxifen-treated women with completely inactive *CYP2D6* enzyme should not have a recurrence risk any higher than women with wild-type *CYP2D6* who are not treated with tamoxifen. Despite this plausible limit, many studies report relative risk estimates substantially higher than 2, which calls into question the validity of their findings. Furthermore, the evidence upon which this limit was derived was known before any of the pharmacogenetic studies was published, and could have been easily incorporated into evidence summaries using Bayesian approaches to meta-analysis.^24^

#### Meta-analyses of CYP2D6 variation and breast cancer recurrence in tamoxifen-treated women

To date there have been 7 meta-analyses, published between 2010 and 2017, of studies reporting the association between impaired CYP2D6 function and breast cancer outcomes in tamoxifen-treated women.^25–31^ Table 1 summarizes the characteristics and results of these meta-analyses, which vary substantially in their quality assessment criteria, population targets, and eligibility criteria. Summary relative risks ranged from 1.25 to 2.08, and no meta-analysis formally incorporated the well-established biological and clinical trial information about the likely effect of CYP2D6 impairment on recurrence risk in tamoxifen-treated women.^23^

### Web-based meta-analysis addresses the limitations of conventional reports

Stakeholders in the CYP2D6/tamoxifen topic area could readily identify faults with some of the CYP2D6/tamoxifen meta-analyses in Table 1. For example, 3 of the 7 meta-analyses excluded studies because they did not report associations as hazard ratios^26,30,31^—which was neither necessary nor advisable^5^—leading to exclusion of at least half the evidence base from their summaries. Disagreement with the approach to a meta-analysis will naturally sow distrust in its results, and readers are unlikely to accept a summary of an evidence base until that summary reflects their own study selection criteria and preferred analytic approach. We contend that an open, web-based meta-analysis would be a more effective and transparent medium for disseminating quantitative summaries of an evidence base—especially in a topic area with appreciable heterogeneity of study findings—than the conventional static-page format. To illustrate, we have built a web-based meta-analysis for the CYP2D6/tamoxifen topic area.

## METHODS

### Search criteria and study selection

We carried out a systematic review of published literature on the association between genetic modification of CYP2D6 activity and breast cancer outcomes in tamoxifen-treated women. Detailed search parameters and study selection criteria are provided in the “Background and search criteria” tab of the web application referenced below. Briefly, we identified relevant studies by searching the MEDLINE database using terms to capture tamoxifen, the CYP2D6 gene/enzyme, breast cancer, and pharmacogenomics. We imposed an English language restriction on the search results. All papers published or presented as abstracts through 31 January 2020 were reviewed. We included studies that compared rates of breast cancer recurrence or breast cancer-specific mortality between women who carry two variant *CYP2D6* alleles and women who carry two wild-type *CYP2D6* alleles. When papers reported findings from duplicate study samples, we retained only the most recent report. From each study we abstracted the first author’s surname, year of publication, PubMed ID, country in which the study population was enrolled, the DNA source used for genotyping (tumour or non-neoplastic tissue), the set of *CYP2D6* alleles genotyped, whether the study population was predominately Asian or Caucasian, and the summary relative estimate of effect and its 95% confidence interval. No studies were excluded based on our assessment of potential bias.

### Development of web-based meta-analysis

We built a web-based meta-analysis platform (available at http://galaxy.med.uvm.edu:3838/thomasahern/2d6meta/ or https://tpahern.shinyapps.io/2d6meta) using the Shiny package for the R statistical programming language, both of which are freely available.^32^ Shiny allows complicated analytic code to be wrapped in a customizable web-based user interface, giving stakeholders without programming expertise an opportunity to alter initial meta-analyses with intuitive point-and-click inputs. Once deployed on a server, Shiny applications are accessible by anyone with an internet-connected computing device (including smartphones and tablets). We built our web-based meta-analysis to comply with reporting recommendations set forth in the “Meta-Analysis of Observational Studies in Epidemiology” (MOOSE) and “Preferred Reporting Items for Systematic Reviews and Meta-Analyses” (PRISMA) statements.^33,34^ It therefore includes comprehensive background information about the CYP2D6/tamoxifen topic, the objectives for the meta-analysis, our literature search criteria, selection criteria from our systematic review, and a tabulation of key information about the studies comprising the evidence base. We included options for conventional^35^ and Bayesian^36^ modeling approaches, the latter of which should be selected by users who wish to incorporate a prior distribution for the association into the meta-analytic summary. Conventional meta-analyses can use either a fixed- or random-effects framework—the latter of which utilizes the DerSimonian-Laird model.^37^ Source data for the meta-analysis and all computer code for the web application are freely available on GitHub (github.com/tpahern/shiny-2d6meta).

### Meta-analysis statistical methods

To illustrate the intended use of web-based meta-analysis by stakeholders in a specific topic area, we generated summaries of the association between CYP2D6 impairment and breast cancer outcome under 3 different modeling approaches (conventional random-effects meta-analysis and 2 Bayesian random-effects meta-analyses with different prior distributions) and with 5 selected subsets of the evidence base (studies measuring the *4 variant in Caucasian populations, studies measuring the *10 variant in Asian populations, studies with RR≤2, studies using tumour-derived DNA, and studies using DNA derived from non-neoplastic tissue). The Bayesian meta-analyses in our example used upper and lower 95% relative risk limits corresponding either to vague or to informative prior distributions for the CYP2D6/recurrence association. The vague prior was defined as a null-centered normal distribution with 95% of relative risks falling between 0.25 and 4 [*i.e.*, ~N(mean=0, variance=0.5)] and the informative prior was defined as a normal distribution centered on a relative risk of 1.41 (halfway between the null and the presumed maximum RR of 2 on the log scale), with variance of 0.031 [*i.e.,* ~N(0.347, 0.03)], corresponding to a prior belief that the relative risk falls between 1 and 2 with 95% certainty. Application users may specify other normal priors for the association by inputting different presumed upper and lower limits for the centered 95% density; the application then reports the mean and variance and plots the resulting distribution for visual reference. A fixed Jeffreys prior for the heterogeneity parameter (τ) was used for all analyses, though users can select other forms.

## RESULTS

Our systematic review identified 36 eligible studies of CYP2D6 impairment in relation to breast cancer recurrence or breast cancer-specific mortality. Studies were heterogeneous with respect to the sets of *CYP2D6* allele(s) assayed, source of DNA for genotyping (tumour vs. non-neoplastic), study populations (European vs. Asian), outcome (BC mortality vs. recurrence), and in their susceptibility to specific biases (*e.g.*, immortal person-time). Characteristics of these studies are reported under the “Study information” tab of the web application.

Table 2 reports results from the set of frequentist and Bayesian meta-analyses described above. Frequentist meta-analysis based on the entire set of eligible studies yielded a summary relative risk of 1.53 (95% CI: 1.25, 1.87). Bayesian meta-analyses of all eligible studies produced similar summary estimates, regardless of whether the vague or informative prior was used. This pattern of summary estimates for the analysis types was consistent within all of the key study subgroups (Asian/*10, Caucasian/*4, non-neoplastic DNA, tumour DNA, and RR≤2). Summary estimates of effect were more heterogeneous when compared between these subgroups. For example, focusing on results from frequentist meta-analysis, the summary RR ranged from 1.19 (95% CI: 0.94, 1.51) in the tumour DNA subgroup to 2.44 (95% CI: 1.48, 4.03) in the Asian/*10 subgroup. These general observations might lead to some additional fine-tuning in study selection. For example, manually trimming the set of Asian/*10 studies to those reporting relative risks ≤2 (that is, within the range of biological plausibility) yields a summary RR of 1.62 (95% CI: 1.13, 2.32), which overlaps substantially with the interval from the frequentist meta-analysis of studies genotyping from tumour DNA. Similarly, starting with the set of studies with RR≤2 and manually removing those that did not genotype the non-functional *4 variant yields a conventional meta-analytic summary estimate of 1.17 (95% CI: 1.02, 1.35). Users may fine-tune the set of contributing studies in this way by checking or unchecking boxes next to each eligible study. For example, a user might uncheck studies that they consider at high risk for bias.

## DISCUSSION

Taken together, the results of the various approaches to the CYP2D6/tamoxifen meta-analysis show that the choice of analytic method (frequentist *vs*. Bayesian model, and vague *vs*. informative prior) made little difference in the ultimate result. The more important difference arose from the choice of study subset. While a moderately strong association in the causal direction was apparent in the Asian/*10 subset, further restriction to studies with biologically plausible effect sizes yielded an attenuated summary association. Our overall impression from this exercise is that the association between reduced CYP2D6 activity and breast cancer recurrence or mortality in tamoxifen-treated women is either null or of small magnitude. Other stakeholders in this topic area are likely to reach similar conclusions when they can change the methods and study selection criteria to their own liking. However, should disagreements persist, the dialogue over factors responsible for the heterogeneous inferences—and therefore the priority features of new research studies—are readily informed by contrasting the disparate sets of studies. Identifying study characteristics most related to differences in meta-analytic results, and therefore to differences in inference, may provide guidance on the most productive design of further research. Our interactive application cannot compensate for a poorly done meta-analysis, and it cannot prevent selective inclusion of studies designed to yield a preordained result. This would constitute a misuse of our application. We note, however, that susceptibility to selective inclusion is not unique to our application, and has been raised previously in this topic area in particular.^38,39^

A key feature of effective systematic review and meta-analysis, set forth in the founding tenets of the Cochrane Collaboration, is the timely and extensive dissemination of evidence to the medical and scientific communities. Dissemination of Cochrane Reviews has morphed in response to technological trends in the decades since Cochrane’s inception—first by print publication, followed by floppy disks, CD-ROMs, and web publication.^6^ Despite this electronic evolution, the fundamental approach to conducting and reporting meta-analyses has not changed from the rigid form imposed by print media. They remain static, unalterable bodies of work whose conclusions depend on judgments made by a small and select group of authors, some of which may not represent the consensus view of other stakeholder groups. We have demonstrated that by linking web-based dissemination with web-based analysis tools, meta-analyses can become an transparent process that involves all stakeholders in a given topic area, which we argue will better serve the aims of evidence synthesis and scientific consensus. It is easy to envision integration of web-based meta-analysis with the concept of “living reviews”—wherein the online meta-analysis would be continually updated with new published studies.^7^ In the meantime, our intention is to refresh our web application annually by adding new eligible studies after repeating our literature search and systematic review. We expect this approach will help to stem the counter-productive “metastasis” of poorly-conducted, redundant, and potentially biased meta-analyses^7^ under the aging static publication paradigm.

## Figures and Tables

**Figure 1.**
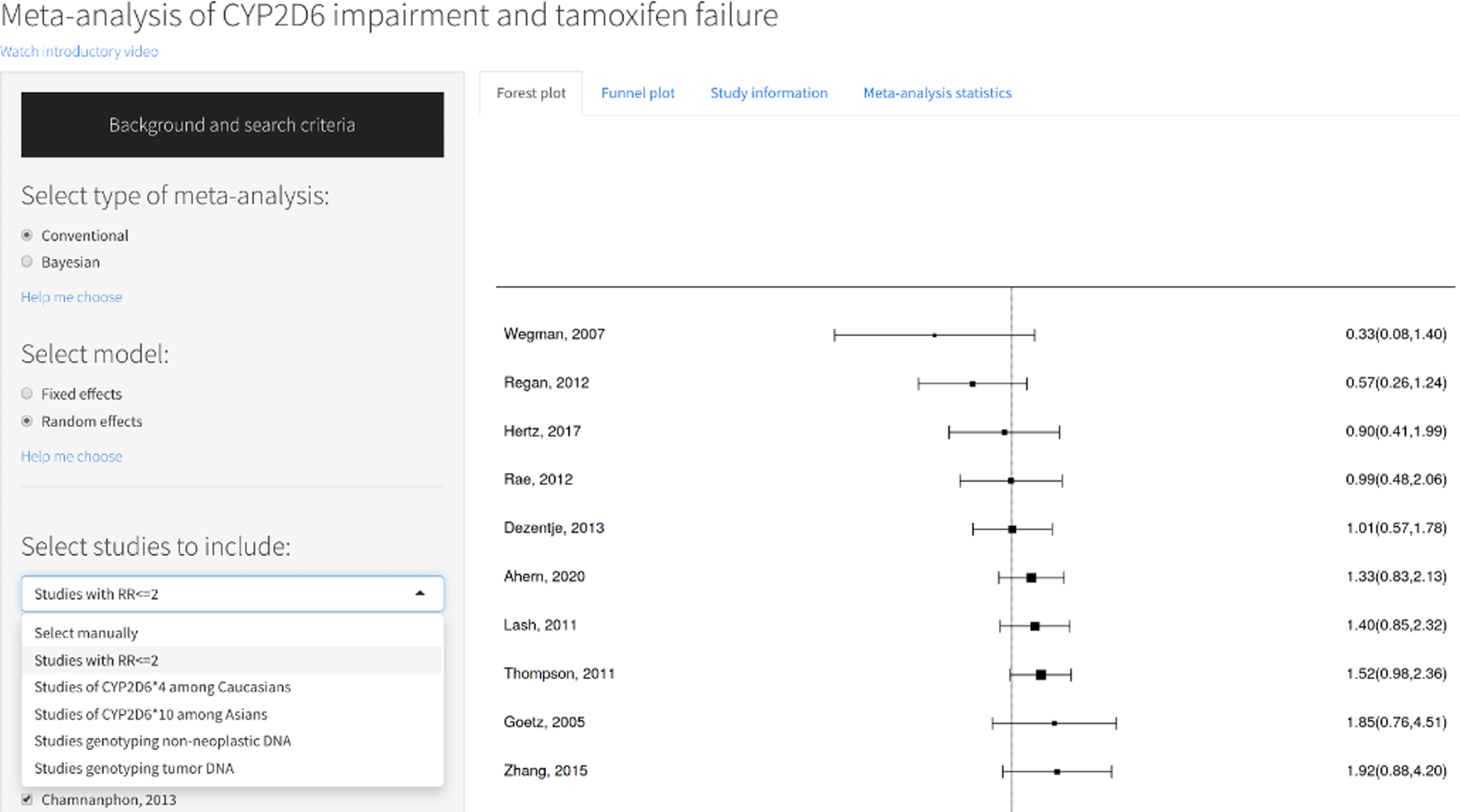
Screen shot of the web-based meta-analysis for the association between genetic impairment of CYP2D6 function and breast cancer recurrence or mortality.

**Table 1.** Summary of published meta-analyses of the association between impaired CYP2D6 function and disease-free survival among breast cancer patients treated with tamoxifen.

Author	Year published	No. studies included	Population(s)	Summary RR (95% CI)	
Seruga	2010	10	All	1.41	(0.94, 2.10)
Lum	2013	25	All	1.34	(1.17, 1.54)
Zeng	2013	20	All	1.37	(1.12, 1.69)
Cronin-Fenton	2014	30	All	2.08	(1.40, 3.10)
Jung	2014	10	All	1.60	(1.04, 2.47)
Province	2014	10	All	1.25	(1.06, 1.47)
Lu	2017	15	Asian	1.79	(1.14, 2.80)

**Table 2.** Results of conventional (frequentist) and Bayesian meta-analyses of the association between impaired CYP2D6 function and disease-free survival among breast cancer patients treated with tamoxifen, under a variety of study selection criteria. Random effects models were used for the conventional analyses. Bayesian models used either vague or informative priors as described in the methods section.

Selection criterion	Meta-analysis type *	Summary RR (95% CI)	
All studies	Conventional	1.53	(1.25, 1.87)
	Bayesian, vague	1.51	(1.23, 1.90)
	Bayesian, informative	1.49	(1.25, 1.80)
Caucasian/*4	Conventional	1.25	(1.06, 1.49)
	Bayesian, vague	1.25	(1.05, 1.50)
	Bayesian, informative	1.28	(1.10, 1.51)
Asian/*10	Conventional	2.44	(1.48, 4.03)
	Bayesian, vague	2.15	(1.25, 3.69)
	Bayesian, informative	1.67	(1.22, 2.24)
RR≤2	Conventional	1.22	(1.05, 1.41)
	Bayesian, vague	1.21	(1.01, 1.42)
	Bayesian, informative	1.25	(1.07, 1.44)
Tumour DNA	Conventional	1.19	(0.94, 1.51)
	Bayesian, vague	1.18	(0.88, 1.55)
	Bayesian, informative	1.26	(1.02, 1.56)
Non-neoplastic DNA	Conventional	1.85	(1.40, 2.46)
	Bayesian, vague	1.81	(1.33, 2.51)
	Bayesian, informative	1.65	(1.31, 2.08)
